# New Drugs and Preparations

**Published:** 1894-05-26

**Authors:** 


### NEW DRUGS AND PREPARATIONS.

[We shall be glad to receive, at our Office, 428, Strand, London, W.C., from the manufacturers, specimens of all new preparations and drugs which may be brought out from time to time.]

### NEURODIN AND THERMODIN.

Professor von Mering (*Therap. Monatsh.*, Dec., 1893) contributes an interesting paper on the action and therapeutical value of several new antipyretics and analgesics. After a valuable clinical and experimental criticism of a number of them, he gives the results obtained with two—neurodin and thermodin—which he has selected as being of practical clinical usefulness. Neurodin is para-oxyphenylacetylethylurethane $C_6H_4 \langle {}^{OCO\ CH_3}_{NHCO\ OC_2H_5}$, a body therefore closely related chemically to phenol, acetanilide, phenylurethane, and other well-known analgesics and antipyretics, but chemically modified with the intention of making it less toxic. It is in colourless, odourless crystals, soluble in 1,400 parts cold water, 140 parts boiling water, readily in alcohol. Rabbits after 45 grains, and dogs after 45 grains daily for a week showed no deleterious effects. In man it was given in typhus and scarlet fevers, pneumonia, pleurisy, and erysipelas as an antipyretic, when it was found that 7 grains lowered the temperature 4 deg. or 5 deg. Fahr. The temperature sank gradually for three or four hours, and then gradually rose again. Sometimes profuse perspiration was present, and occasionally shivering, but never any severer symptoms, although in one case a skin eruption occurred. The author is of opinion that its antipyretic value is not greater than that of many other substances. He lays great stress, however, on its anti-neuralgic properties. He gave usually 15 grains, and the action is well pronounced within half an hour. He gave it in all kinds of neuralgic cases up to 60 grains, or even 90 grains daily. With these larger doses faintness and sleepiness occurred. On comparing it with phenacetin, he found that sometimes the one, sometimes the other, was most successful, and advises its use as an alternative in cases where the former fails. It is best prescribed simply as powder.

Thermodin is a somewhat more complex body of the same nature, para-ethoxyphenylacetylethylurethane $C_6H_4 \langle {}^{OC_2H_5}_{NCOCH_3\ COOC_2H_5}$, which crystallises in white, odourless, and almost tasteless crystals, scarcely soluble in water. It was tried in fifty cases, including typhoid, pneumonia, pleurisy, influenza, &c., with the result that von Mering characterises it as the best antipyretic yet introduced. After 7 to 10 grain doses the temperature fell about 3 deg. to 4 deg. Fahr., the action beginning in about one hour, and reaching its maximum in about four hours. There is moderate sweating, slowing and higher tension of the pulse, very seldom rigors. In one case a skin eruption something like measles occurred.

It is best given in powder form, and for phthisical cases somewhat smaller doses are recommended. In influenza, besides lowering the temperature it greatly modified the subjective symptoms, but was not found to be of especial value as an analgesic.

### DUBOISINE SULPHATE.

Rabow[1] discusses the chemical composition and action of commercial duboisine sulphate. It is an alkaloid derived from the Pituri plant (Duboisia Hopwoodii), the leaves of which are used and smoked by the Australian aborigines as a stimulant narcotic. It seems to be a mixture of several alkaloids closely resembling atropine, hyoscyamine, hyoscine, and other atropaceous alkaloids. Its action has a generic resemblance to all these, but differs in intensity and in minor details. It acts more rapidly and strongly as a mydriatic than hyoscine, but less powerfully as a sedative or hypnotic. The sulphate forms a yellowish amorphous powder, readily soluble in water, and quite unirritating. It is extremely active, and 1-65 grain has caused quickening of respiration and pulse, hallucinations, and some degree of delirium. Very minute doses dilate the pupil in about five minutes, but it has been almost completely discarded from ophthalmic practice on account of its tendency to cause toxic symptoms unless used with the extremest care. In checking the night-sweats of phthisis it is inferior to atropine, and not superior to it in controlling excessive salivation. In doses of 1-120 to 1-60 grain subcutaneously it has proved useful in exophthalmic goitre, in paralysis agitans, and in a case of congenital nystagmus. Rabow has found it valueless in epilepsy, hysteria, and chorea.

Ostermayer,[2] as a sedative and hypnotic in lunacy practice, giving it in 1-65 to 1-20 grain doses, found it very effective, and never observed any poisonous effects. Rabow has given it about 400 times, and finds that 1-120 to 1-60 grain has a sedative effect while 1-30 grain is hypnotic. The latter dose corresponds in effect to about 30 grains chloral hydrate. It very soon loses its effect, but regains it again if the drug be intermitted for a few days. A combination with chloral hydrate acts well. On the whole he regards it as less dangerous than hyoscine, but unpleasant symptoms are not uncommon, such as a feeling of fatigue, lightness in the head, dryness of throat, dilatation of pupils, and rapid pulse. The sleep caused by it is not very refreshing, and he does not recommend it strongly for use as a hypnotic, but is of opinion that it is a valuable sedative in excited mental conditions.

Montyel[3] also finds it analogous in its effects to hyoscine and hyoscyamine. Its calmative action was very marked in general paralysis, mania, and acute melancholia, but he found that it was very apt to cause digestive disturbances and emaciation.

Albertoni[4] used duboisine sulphate in a case of aggravated hystero-epilepsy with success after many other methods of treatment had been without result. He points out, however, that it is a body of uncertain composition, as we find it in commerce, and that hence one may not get equally good results with different samples.

[1] Therap. Monatsh., 1893, 410. [2] Allgem. Ztschr. f. Psychiat. xlvii., 1890. [3] Archiv de Neurol, Sept., 1893. [4] Therap. Monatsh., 1893, 409.

